# Exploring Dried Blood Spot Cortisol Concentrations as an Alternative for Monitoring Pediatric Adrenal Insufficiency Patients: A Model-Based Analysis

**DOI:** 10.3389/fphar.2022.819590

**Published:** 2022-03-17

**Authors:** Viktoria Stachanow, Uta Neumann, Oliver Blankenstein, Davide Bindellini, Johanna Melin, Richard Ross, Martin J. Whitaker, Wilhelm Huisinga, Robin Michelet, Charlotte Kloft

**Affiliations:** ^1^ Department of Clinical Pharmacy and Biochemistry, Institute of Pharmacy, Freie Universitaet Berlin, Berlin, Germany; ^2^ Graduate Research Training Program PharMetrX, Berlin, Germany; ^3^ Pediatric Endocrinology, Charité-Universitätsmedizin, Berlin, Germany; ^4^ Labor Berlin, Charité Vivantes GmbH, Berlin, Germany; ^5^ Diurnal Limited, Cardiff, United Kingdom; ^6^ Institute of Mathematics, Universität Potsdam, Potsdam, Germany

**Keywords:** adrenal insufficiency, cortisol, dried blood spots, pediatrics, pharmacokinetics, binding, association, red blood cells

## Abstract

Congenital adrenal hyperplasia (CAH) is the most common form of adrenal insufficiency in childhood; it requires cortisol replacement therapy with hydrocortisone (HC, synthetic cortisol) from birth and therapy monitoring for successful treatment. In children, the less invasive dried blood spot (DBS) sampling with whole blood including red blood cells (RBCs) provides an advantageous alternative to plasma sampling. Potential differences in binding/association processes between plasma and DBS however need to be considered to correctly interpret DBS measurements for therapy monitoring. While capillary DBS samples would be used in clinical practice, venous cortisol DBS samples from children with adrenal insufficiency were analyzed due to data availability and to directly compare and thus understand potential differences between venous DBS and plasma. A previously published HC plasma pharmacokinetic (PK) model was extended by leveraging these DBS concentrations. In addition to previously characterized binding of cortisol to albumin (linear process) and corticosteroid-binding globulin (CBG; saturable process), DBS data enabled the characterization of a linear cortisol association with RBCs, and thereby providing a quantitative link between DBS and plasma cortisol concentrations. The ratio between the observed cortisol plasma and DBS concentrations varies highly from 2 to 8. Deterministic simulations of the different cortisol binding/association fractions demonstrated that with higher blood cortisol concentrations, saturation of cortisol binding to CBG was observed, leading to an increase in all other cortisol binding fractions. In conclusion, a mathematical PK model was developed which links DBS measurements to plasma exposure and thus allows for quantitative interpretation of measurements of DBS samples.

## Introduction

Congenital adrenal hyperplasia (CAH) is a group of rare autosomal recessive diseases, which are characterized by largely decreased or absent cortisol biosynthesis. In 90–95% of cases, a deficiency of the 21-hydroxylase enzyme is the cause for CAH (Podgórski et al., 2018; Balsamo et al., 2020). A major complication in patients is an adrenal crisis which may even lead to death. Other symptoms of CAH include virilization, hirsutism, premature adrenarche, and premature ending of longitudinal growth due to an overproduction of androgens and possible life-threatening electrolyte imbalance due to the underproduction of mineralocorticoids (Merke and Bornstein, 2005).

The treatment of CAH requires life-long cortisol replacement therapy. The recommended glucocorticoid for pediatric CAH patients is hydrocortisone (HC, name of synthetic cortisol) due to its short half-life and lower risk for adverse events (Oprea et al., 2019). To mimic the circadian rhythm of cortisol biosynthesis, oral administration of 10–15 mg/m^2^ hydrocortisone daily is recommended, divided into two to three doses, and with the highest dose in the morning (Kamoun et al., 2013; Khattab and Marshall, 2019; Dabas et al., 2020). It is essential to monitor cortisol replacement in CAH patients frequently and adjust dosages according to the patients’ individual needs, based on the body surface area, laboratory parameters, and symptoms evaluation (Bornstein et al., 2016) as too high or too low cortisol exposure can cause adverse events, such as Cushing’s syndrome, and or lead to an adrenal crisis (Merke and Bornstein, 2005).

Dried blood spot (DBS) samples have been used since the 1960s to perform newborn screenings for diseases such as phenylketonuria (Moat et al., 2020). Currently, because of technological development allowing for more specific and sensitive specimen analysis, DBS has been exploited to monitor CAH patients (Moat et al., 2020). DBS sampling consists of dropping small volumes of whole blood drops (approximately 20 µl) collected via a fingerpick on a cellulose-based sampling paper. Therefore, this sampling procedure is simpler and less invasive than traditional plasma sampling. It is thus of great advantage for the pediatric population because of their vulnerability and limited blood volume (Qasrawi et al., 2021). DBS sampling provides additional benefits such as higher analyte stability, allowing storage at room temperature, and easy transportation (Edelbroek et al., 2009; Wilhelm et al., 2014).

To evaluate the applicability of DBS sampling, it is essential to understand the relationship between cortisol DBS concentrations, and plasma concentrations. Cortisol has complex PK with saturable binding to corticosteroid-binding globulin (CBG) and linear binding to albumin which previously has been identified using a nonlinear mixed-effects (NLME) HC PK model [(Melin et al., 2017; Michelet et al., 2020)]. Moreover, cortisol is known to associate with RBCs (Lentjes and Romijn, 1999). This is of special interest for interpreting DBS samples as these are whole blood samples containing RBCs. The aim of this analysis was to explore and quantify the relationship between venous DBS cortisol concentrations and plasma cortisol concentrations by characterizing the association of cortisol with RBCs; which is the first step towards the use and interpretation of DBS samples for monitoring pediatric CAH patients.

## Methods

### Data

A previously published NLME HC PK model based on cortisol plasma data from healthy adults and pediatric patients (Melin et al., 2017; Michelet et al., 2020) served as the starting point for our analysis. The model leveraged data from 1) rich plasma sampling (*n* = 1,482 total cortisol concentrations) in a phase 1 study (Whitaker et al., 2015) with 30 healthy adult subjects, whose cortisol biosynthesis was suppressed with dexamethasone, and who received a single dose of 0.5 mg up to 20 mg of the pediatric HC formulation Alkindi^®^ (hydrocortisone granules in capsules for opening) (Diurnal Europe B.V., Netherlands).

Additionally, the model leveraged 2) sparse phase 3 cortisol plasma data from 24 pediatric adrenal insufficiency (AI) patients receiving their regular HC-morning dose of Alkindi^®^, ranging from 1 to 4 mg (Neumann et al., 2018; Melin et al., 2020; Michelet et al., 2020). The pediatric patients were divided into three different cohorts according to their age groups: Young children (*n* = 12, 2–6 years), infants (*n* = 6, 28 days-2 years), and term neonates (*n* = 6: 0–28 days). The pediatric total cortisol plasma concentrations were measured prior to dose and 1 and 4 h post-dose in all cohorts. In neonates and infants, the sampling was ethically limited to these 3 times due to the lower total blood volume, whereas in the children cohort, blood sampling at 2 additional times between 30 and 90 h post-dose was allowed as well as at time to C_min_ (t_min_).

To expand this model, simultaneously collected venous total cortisol DBS samples, obtained from the pediatric patients in the phase 3 study, were included. Both total cortisol concentrations in plasma (Whitaker et al., 2015; Melin et al., 2017; Michelet et al., 2020) and in DBS (*n* = 106 each) were quantified by liquid chromatography with tandem mass spectrometry detection (LC-MS/MS). Linearity, accuracy, and precision were tested for DBS cortisol quantification with the respective acceptance criteria being met according to the guideline on bioanalytical method validation of the European Medicines Agency (European Medicines Agency, 2012).

### Graphical Evaluation of Plasma Versus DBS Cortisol Concentrations

The relationship between the pediatric total cortisol concentrations in plasma and DBS was graphically evaluated based on concentration–time profiles, plotting plasma versus DBS cortisol concentrations and graphically investigating the plasma/DBS ratio as a function of the cortisol concentration, and the cortisol concentration dependency of the plasma/DBS cortisol concentration ratio. The graphical analysis was performed using R (3.6.0) and R Studio (1.3.1056) (R Core Team, 2019; RStudio Team, 2020).

### Pharmacokinetic Model Development and Evaluation

The previously published NLME HC PK model, based on adult and pediatric plasma cortisol data, was a two-compartment PK model describing saturable absorption (Michaelis–Menten type) and a plasma protein binding model considering both nonlinear binding to CBG and linear binding to albumin. An underlying constant cortisol baseline was estimated for the adult data, whereas for the pediatric cortisol data, the baseline was modeled using the individual measured pre-dose concentration. For baseline cortisol concentrations below the lower limit of quantification (LLOQ), a baseline concentration was estimated with the same interindividual variability as the observed pre-dose concentrations above LLOQ (Melin et al., 2020; Michelet et al., 2020).

Body weight was included as an influential factor using theory-based allometric scaling with fixed exponents of 0.75 and 1 on the clearance parameters (CL and Q) and on the volumes of distribution (V_c_ and V_p_), respectively, to account for differences in the body size within the pooled dataset. No other covariates besides body weight were evaluated in the structural plasma PK model. Interindividual variability (IIV) was modeled, assuming the structural model parameters to follow a log-normal distribution, and residual unexplained variability (RUV) was modeled following a proportional residual error model (Melin et al., 2020; Michelet et al., 2020).

Based on this PK model structure and modeling approach, the published HC PK model was further developed by extending the underlying data with the pediatric DBS cortisol concentrations. Implemented cortisol binding processes were extended by the association of cortisol with RBCs which were all assumed to contain hemoglobin. For the model development, NONMEM (7.4.3, ICON, Dublin, Ireland, Development Solutions, Ellicott City, MD, United States) and Perl-speaks-NONMEM (3.4.2, Uppsala University, Uppsala, Sweden), embedded in the workbench Pirana (version 2.9.6), were used (Bauer, 2010; Keizer et al., 2013). The appropriateness of the PK model was evaluated based on standard model diagnostics, for example, the difference of the objective function value (dOFV, best fit = maximum likelihood = minimum OFV) and goodness-of-fit (GOF) plots (Mould and Upton, 2012, 2013). Model performance was evaluated using visual predictive checks (VPCs, n = 1,000 simulations) (Bergstrand et al., 2011) (see the Supplementary Material) and sampling importance resampling (SIR, with 1,000, 1,000, 1,000, 2,000, and 2,000 samples and 200, 400, 500, 1,000, and 1,000 resamples) (Dosne et al., 2017).

### Simulation of Cortisol Binding Species

The final and evaluated PK model allowed simulating the fractions of the three different binding/association species of cortisol (specific binding to CBG, non-specific binding to albumin, and non-specific association with RBCs) and the unbound cortisol fraction. One individual representing the children/infants age group and one individual representing neonates were virtually dosed with 7 mg HC each; the concentrations of the binding species and cortisol whole blood concentrations were simulated over 6 h [deterministic simulations using NONMEM (7.4.3)].

## Results

### Data

Of the pediatric plasma and DBS concentrations, 17.9% (*n* = 19 of 106, LLOQ = 14.1 nmol/L) and 0.94% (*n* = 1 of 106, LLOQ = 1.8 nmol/L) were below the LLOQ, respectively. All adult total cortisol plasma concentrations were above the LLOQ. As in the previously published model, all BLQ observations were discarded so that 87 pediatric plasma samples and 105 pediatric DBS samples remained for the subsequent graphical analysis, modeling, and simulation analysis.

### Graphical Evaluation of Plasma Versus DBS Cortisol Concentrations

**FIGURE 1:**
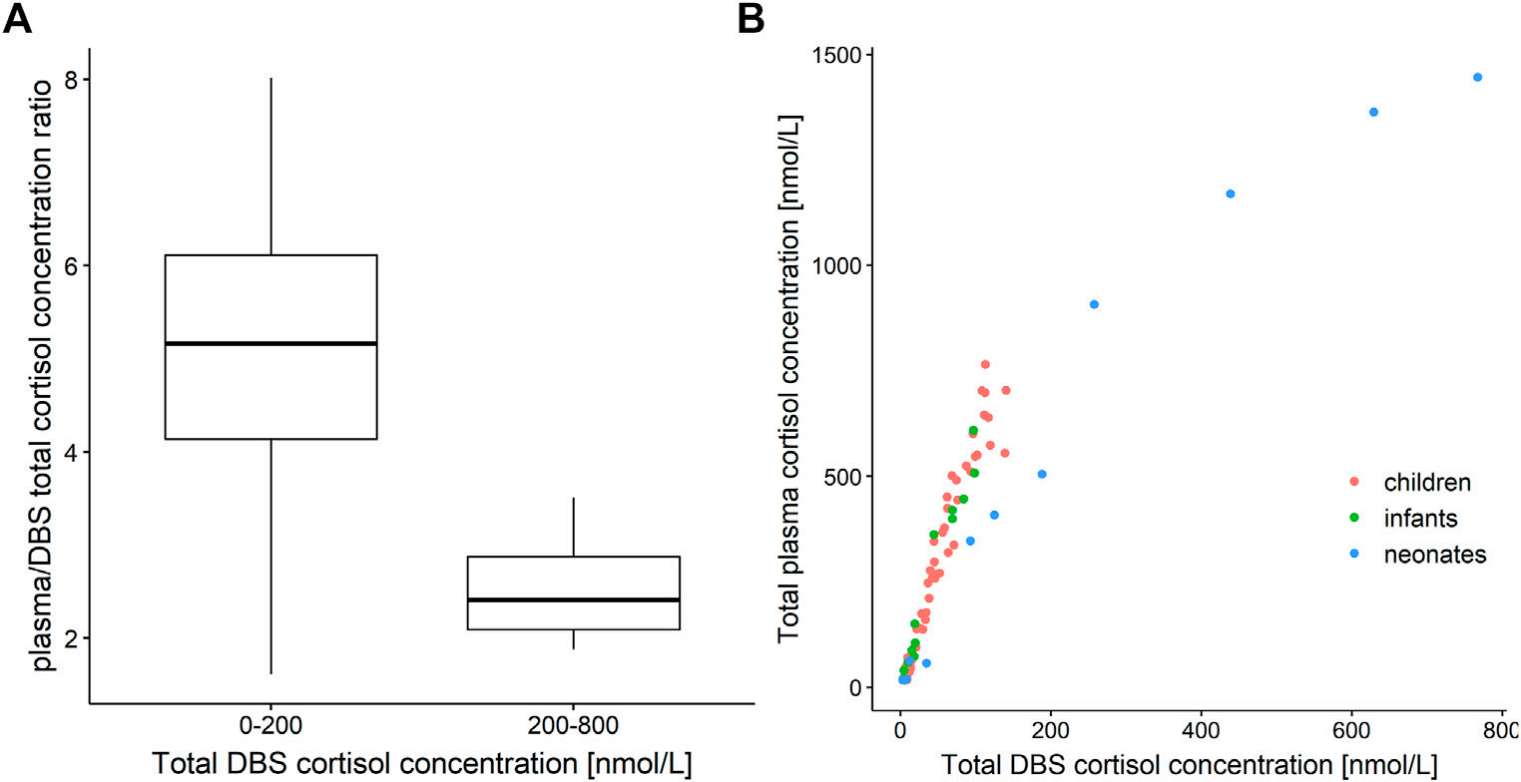
**(A)** Boxplot of plasma/dried blood spot (DBS) cortisol concentration ratio versus cortisol DBS concentration ranges of 0–200 nmol/L (*n* = 83) and 200–800 nmol/L (*n* = 4). **(B)** Total cortisol concentration in plasma versus total cortisol concentration in dried blood spots (DBS). Red: children, green: infants, blue: neonates.

Measured plasma cortisol concentrations were considerably higher than DBS cortisol concentrations with ratios with a very high variability, ranging from approximately 2 to 8 (Figure 1A). The relationship between total cortisol concentrations in plasma and DBS was nonlinear (Figure 1B), where the plasma/DBS cortisol concentration ratio decreased with higher cortisol concentrations, reaching the lowest ratio at the highest concentrations. Regarding the ratio and slope, the data shown in Figure 1B could be divided into 2 groups, with DBS concentrations ranging from 0 to 200 nmol/L and from 200 to 800 nmol/L. When observing cortisol DBS concentrations from 0 to 200 nmol/L (*n* = 83, Figure 1A), the plasma/DBS cortisol concentration ratio ranges widely from 1.62 up to 8.01, with a median of 5.17. With higher concentrations (*n* = 4), which were only observed in the neonatal age group, the concentration ratio decreases to a median value of 2.41 (range 1.88–3.51). The comparison of DBS concentration ranges from 0 to 100 nmol/L and from 100 to 200 nmol/L showed no significant difference in plasma/DBS cortisol concentration ratios.

**FIGURE 2:**
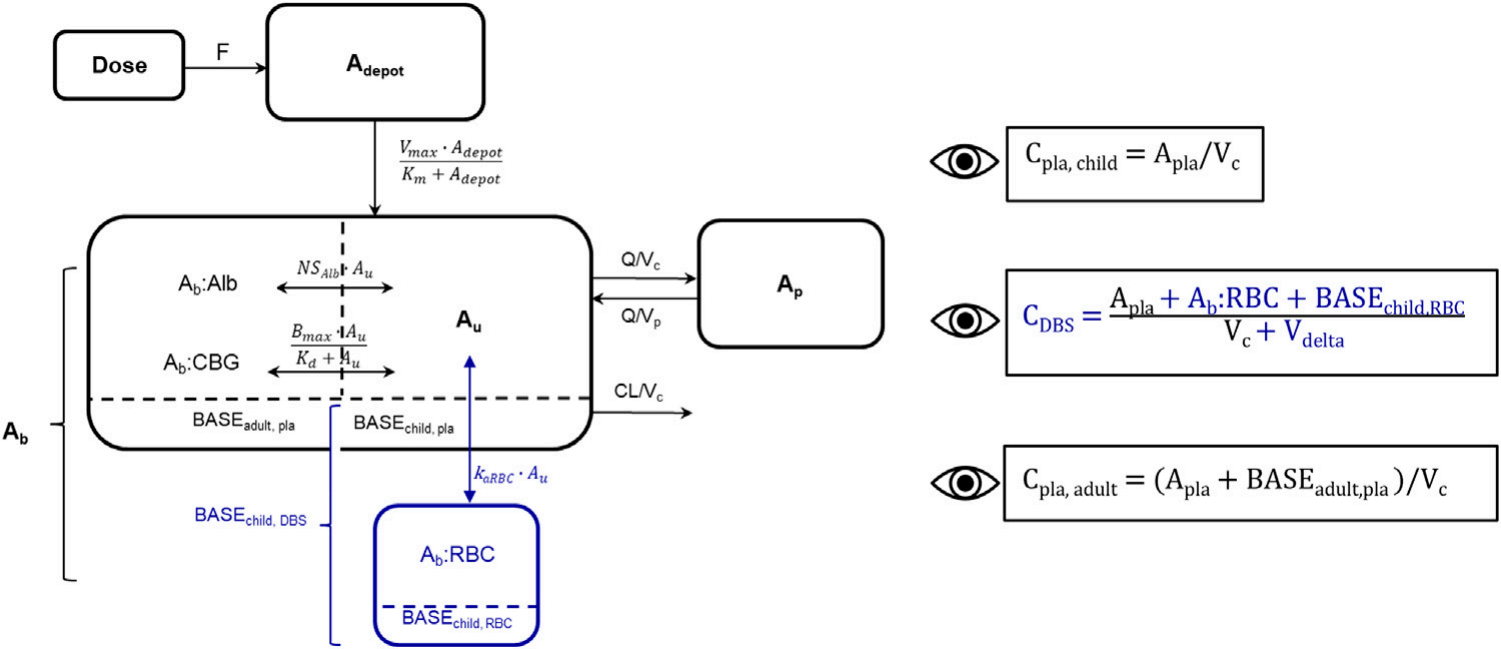
Schematic representation of developed cortisol PK model including adult plasma data and pediatric plasma and dried blood spot (DBS) data, blue: new DBS-related compartments/parameters/data. Bioavailability (F), amount in depot compartment (A_depot_), maximum absorption rate (V_max_), amount in depot compartment resulting in half of V_max_ (K_m_), amount bound (A_b_), amount bound to albumin (A_b_:Alb), amount associated to red blood cells (A_b_:RBC), unbound amount (A_u_), amount bound to corticosteroid-binding globulin (A_b_:CBG), linear non-specific parameter for albumin binding (NS_Alb_) and association to red blood cells (k_aRBC_), maximum binding capacity (B_max_), equilibrium dissociation constant (K_d_), intercompartmental clearance (Q), peripheral volume of distribution (V_p_), cortisol plama baseline of dexamethasone suppressed healthy adults (BASE_adult, pla_), cortisol plasma baseline of children (BASE_child, pla_), cortisol DBS baseline of children (BASE_child, DBS_), cortisol associated to red blood cells at baseline in children (BASE_child, RBC_). The dashed line divides the central compartment into the A_b_ and A_u_ subcompartments, respectively. Eyes next to equations indicate observed concentrations: Pediatric plasma (C_pla, child_) and DBS (C_DBS_) concentrations, adult plasma concentrations (C_pla, adult_), volume of red blood cell compartment (V_delta_).

### Pharmacokinetic Model Development and Evaluation

Since the association of cortisol with RBCs is described as a linear process in the literature (Lentjes and Romijn, 1999), a linear association constant K_aRBC_ was estimated. An additional compartment describing cortisol bound to RBCs was implemented, and an additional apparent volume (V_delta_) was estimated, with the whole blood volume being defined as the sum of V_c_ and V_delta_. Similarly, to the pediatric plasma cortisol baseline (BASE_child, pla_), a pediatric DBS cortisol baseline (BASE_child, DBS_) was implemented, and based on pre-dose observations or was estimated if no observation was present. BASE_child, pla_ was used as an initial value of the central plasma concentration, whereas BASE_child, RBC_ represented cortisol bound to RBCs at baseline and was described in amounts (nmol) as A_BASERBC_ = A_BASEchild, DBS_–A_BASEchild, pla_ (Figure 2). The underlying model equations can be found in the Supplementary Material.

Since higher cortisol concentrations, leading to relatively higher cortisol DBS concentrations, were only observed in the neonatal cohort, “age group” was evaluated as a dichotomous categorical covariate, that is, the influential factor (“children/infants” and “neonates”) on the estimated V_delta_. The inclusion of this covariate resulted in −47.06 dOFV and explained more than two-thirds (69.1%) of the interindividual variability (IIV) of V_delta_ (before: 196% CV, after: 60.6% CV). Age was tested as a covariate on V_delta_, with exponential and fractional changes from the median age, and resulted in an OFV drop of −23.0 and of −6.3, explaining 20 and 27% of the V_delta_ IIV, respectively. Given the higher reduction of IIV and the limited neonatal data, the two age groups were chosen and kept in the model as the simplest and thus most appropriate covariate.

Table 1 shows the parameter estimates and the SIR medians and 95% confidence intervals (CI) of the final PK model including adult plasma cortisol data and pediatric plasma and DBS cortisol data. The resulting V_delta_ for children/infants was 11.1 L compared to 1.05 L in neonates, corresponding to 0.82 L/kg and 0.29 L/kg for children + infants and for neonates, respectively. The linear binding/association parameters of cortisol to albumin (NS_Alb_) and RBCs (K_aRBC_) resulted in 4.15 and 6.62, respectively. The predominant binding partner was CBG, with a fixed K_d_ of 9.71 nmol/L, indicating the unbound cortisol concentration at 50% of B_max_. The estimates for the pediatric plasma and DBS cortisol baselines were 9.41 nmol/L and 4.22 nmol/L, respectively.

**TABLE 1:** Parameter estimates with sampling importance resampling (SIR) median and 95% confidence intervals (CI) of developed cortisol pharmacokinetic (PK) model including adult plasma data and pediatric plasma and dried blood spot (DBS) data.

Parameter	SIR median [95% CI]
Structural model	
CL [L/h]	400 [289–549]
V_c_ [L]	10.6 [7.99–14.0]
Q [L/h]	160 [90.4–268]
V_p_ [L]	124 [80.7–178]
K_m_ [nmol]	4,810*
V_max_ [nmol/h]	21,388 [13,888–31,463]
F [−]	1*
K_d_ [nM]	9.71*
NS_Alb_ [−]	4.15*
K_aRBC_ [−]	6.62 [1.95–13.4]
V_delta, children+infants_ [L]	11.1 [7.05–18.8]
V_delta, neonates_ [L]	1.05 [0.50–1.80]
BASE_adult, pla_ [nM]	15.2 [11.1–20.7]
BASE_child, pla_ [nM]	9.41 [3.32–16.7]
BASE_child, DBS_ [nM]	4.22 [1.10–7.60]
Interindividual variability	
ωCL, %CV	25.8 [14.9–35.8]
ωK_m_, %CV	55.7 [31.1–75.5]
ωV_max_, %CV	46.5 [30.1–65.5]
ωF, %CV	36.1 [20.4–49.4]
ωV_delta_, %CV	43.4 [27.5–62.2]
ωBASE_adult,pla_, %CV	35.3 [23.5–47.4]
ωBASE_child,pla and DBS_, %CV	131.1*
Residual variability	
σ_exp_ [CV%]	14.4 [13.2–16.0]

Clearance (CL), central volume of distribution (V_c_), intercompartmental clearance (Q), peripheral volume of distribution (V_p_), amount in depot compartment resulting in half of Vmax (Km), maximum absorption rate (V_max)_, bioavailability (F), equilibrium dissociation constant (K_d_), linear non-specific parameter for albumin binding and association to red blood cells (NS_Alb_ and k_aRBC_), volume of red blood cell compartment (V_delta_), plasma cortisol baseline in adults (BASE_adult, pla_), plasma cortisol baseline in children (BASE_child, pla_), dried blood spot cortisol baseline in children (BASE_child, DBS_). For BASE_child, pla_ and BASE_child, DBS_ a common interindividual variability was fixed, residual variability was estimated as an additive error on a logarithmic scale.

*fixed parameter.

The 95% CIs for the parameter estimates resulting from the SIR show good precision of the parameter estimates. Standard model evaluations, that is, GOF plots and VPCs showed that the adult and pediatric plasma and the pediatric DBS concentrations were adequately described by the final PK model (see the Supplementary Material).

### Simulation of Cortisol Binding Species

**FIGURE 3:**
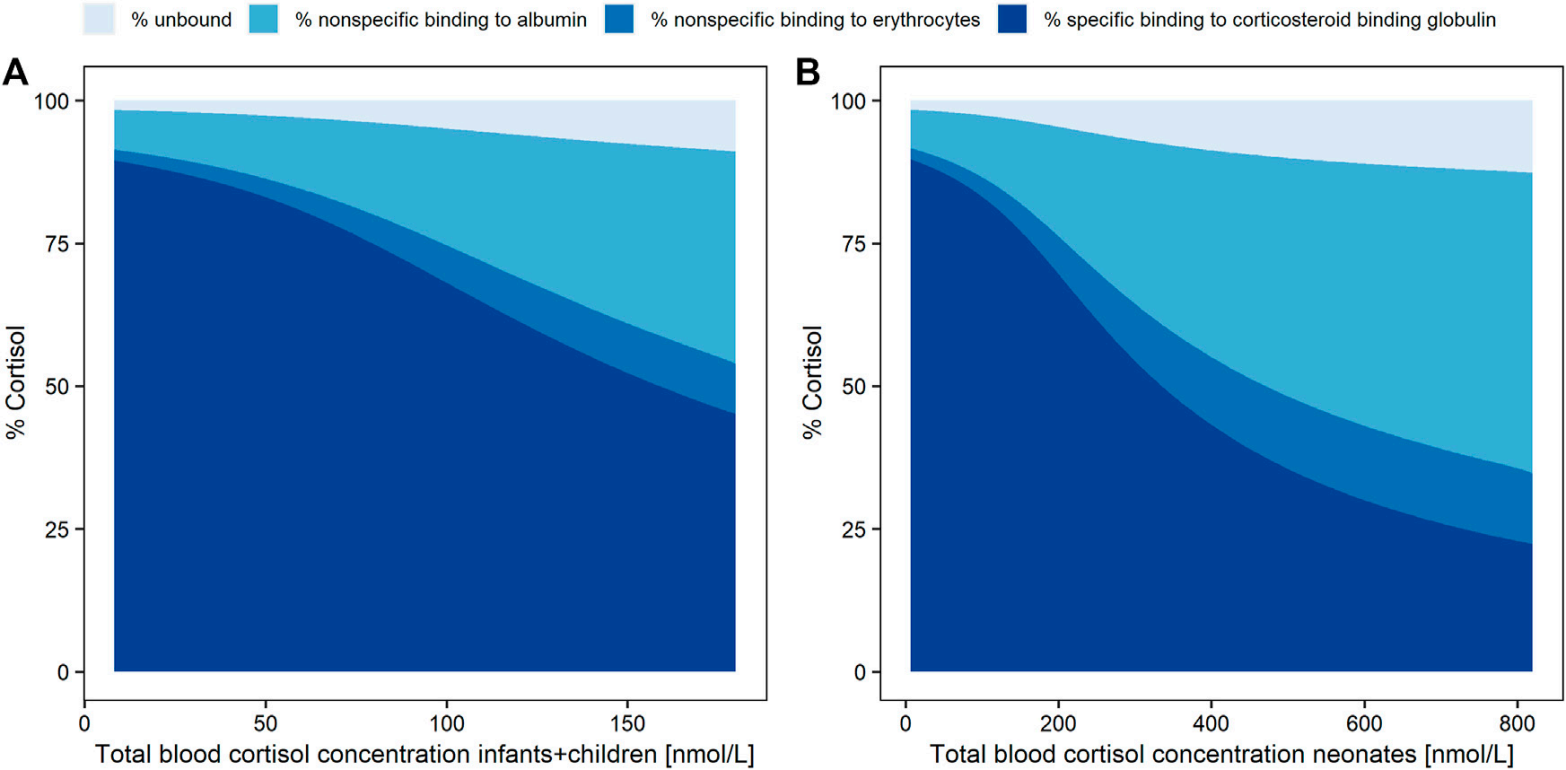
Simulated cortisol concentration fractions (%) as unbound (pale blue), with nonspecific linear binding to albumin (light blue), nonspecific linear binding to red blood cells (middle blue) or specific nonlinear binding (dark blue) to corticosteroid binding globulin, over total whole blood (dried blood spot) concentration (LLOQ = 1.8 nmol/L to Cmax) in infants and children **(A)** and neonates **(B)**.

Simulated whole blood cortisol concentrations of children/infants (Figure 3A) and of neonates (Figure 3B) were based on the PK model with the two different V_deltas_ of the respective age groups based on a dose of 7 mg HC. The fractions of the simulated cortisol species (%) against the whole blood concentration demonstrated the substantial decrease in the cortisol fraction bound to CBG with higher total cortisol concentrations due to the saturation of the binding process. The fraction bound to CBG decreased from approximately 90% at 1.8 nmol/L (LLOQ; i.e., shortly after drug intake) to 45 and 22% at the highest simulated whole blood cortisol concentrations for children/infants (180 nmol/L = Cmax, maximum concentration) and neonates (820 nmol/L = Cmax), respectively. Consequently, a substantial increase was observed for the fraction unbound (children/infants: from 1.7 to 9.0%; neonates: from 1.6 to 13%), the fraction bound to albumin (children/infants: from 7.0 to 37%; neonates: from 6.8 to 53%), and the fraction associated with RBCs (children/infants: from 1.9 to 8.3%; neonates: from 1.9 to 12%).

## Discussion

Leveraging cortisol concentrations from adult plasma data and pediatric plasma and DBS data, we successfully established a quantitative link between pediatric total plasma cortisol concentrations and pediatric total DBS cortisol concentrations by extending a published NLME HC PK model based on adult and pediatric plasma cortisol data with pediatric DBS concentrations.

The inclusion of the whole blood DBS cortisol data into the model allowed to quantitatively characterize cortisol association with RBCs by a linear, non-specific association process, in addition to cortisol binding to CBG, and to albumin. Since the linear association of cortisol with RBCs was only described in adults (Lentjes and Romijn, 1999) and generally a saturation of this process should not be expected due to the abundance of RBCs in all age groups, we assumed that these findings also apply to children. A corresponding RBC-associated cortisol compartment was added to the PK model and RBC volumes were successfully estimated for the neonatal age group and for children/infants. With the age group implemented as a covariate on V_delta_, the V_delta_ estimate (1.05 L) for neonates was considerably lower than the estimated V_delta_ for children/infants (11.1 L) (Table 1) and thereby, as an apparent volume of distribution, accounting for the lower plasma/DBS concentration ratio which was observed for neonates.

The estimates for the pediatric baselines in plasma and DBS were reasonably low and close to respective LLOQs. Overall, the inclusion of the pediatric cortisol DBS data barely changed the plasma-related parameter estimates, which were taken from the previous model, whereas the additional DBS-related parameters resulted in plausible estimates.

For a better comparison of K_aRBC_ (6.62) with the other two binding parameters K_d_ (9.71 nmol/L) and NS_Alb_ (4.15), the PK model was applied to simulate the fractions of cortisol bound to CBG, to albumin, and associated with RBCs. As already expected from the graphical analysis, with increasing cortisol concentrations the ratio between cortisol plasma and DBS concentrations decreased. The simulation results supported this finding, due to the saturation of the nonlinear binding between cortisol and CBG and thus higher availability of unbound cortisol to be associated with RBCs. The simulated fraction of cortisol bound to CBG decreased by 75% in neonates (from 90 to 22%) compared to a decrease of 50% in children/infants (from 90 to 45%). Consequently, a considerably higher amount of free cortisol became available for binding to albumin or association with RBCs. At the highest simulated whole blood cortisol concentrations, 45% more cortisol is associated with RBCs in neonates (12% at 820 nmol/L Cmax) compared to children/infants (8.3% at 180 nmol/L Cmax). These considerably different simulated fractions of cortisol associated with RBCs could partly explain the highly variable plasma/DBS concentration ratios which were observed in the graphical analysis for the two age groups. The higher cortisol concentrations were only observed in neonates due to a higher dose relative to body weight in the phase 3 trial, which was mimicked in the simulations by dosing both age groups with the same HC dose. Since the simulations were deterministic, that is, did not include interindividual variability, simulations with 4 mg (Supplementary Figure S3), which was the maximum single dose given in the phase 3 study, resulted in maximum concentrations lower than the ones observed in the phase 3 study (767.5 nmol/L and 141 nmol/L for neonates and children + infants, respectively). Thus, to ensure simulated total blood cortisol concentrations representing the full range of observed concentrations, the simulation dose was increased to 7 mg. It was assumed that the plasma/DBS concentration ratio depends on the overall cortisol concentration. The high cortisol concentrations observed in neonates were the result of a relatively higher HC dose given in the phase 3 study to avoid underdosing in this highly vulnerable cohort. To further explain the high variability observed in the plasma/DBS concentration ratio, more data are required to investigate whether, besides the concentration dependency, there is also, for example, an age dependency for the ratio.

The graphical investigation of the plasma/DBS cortisol concentration ratio leads to the conclusion that with the observed DBS cortisol concentrations ranging from 0 to 200 nmol/L and from 200 to 800 nmol/L, the corresponding plasma cortisol concentrations are higher than DBS concentrations by a factor of 5 and 2.5, respectively. However, this finding should be confirmed with a richer dataset, especially in the higher concentration range where only four neonatal samples were available, before being considered as a rule of thumb when DBS cortisol sampling is used for clinical monitoring in pediatric adrenal insufficiency patients.

As the cortisol concentration data from the pediatric population were sparse in general, the PK model should be re-evaluated with more plasma and DBS cortisol data to confirm the conclusions of our current analysis. A regression equation based on simulations from the updated PK model could then be identified to enable the calculation of plasma cortisol concentrations from measured DBS cortisol concentrations, opening the opportunity to routinely use and interpret DBS sampling for monitoring this vulnerable patient population. As this analysis is based on data from patients aged from 0 to 6 years, the applicability of the PK model to children older than 6 years can be investigated in future with respective available data. The PK in adolescents aged 12 to 18 years can be assumed to be similar to adult HC PK and binding kinetics as it was found in published pharmacokinetic analyses (Bonner et al., 2021).

Furthermore, the DBS data used in this analysis were venous whole blood concentrations, whereas in clinical practice capillary whole blood is obtained for DBS sampling. It is therefore important to re-evaluate the comparability of venous and capillary whole blood cortisol concentrations. Moreover, the underlying mechanism behind cortisol being associated with RBCs is still unknown, and it should be investigated if cortisol associated with RBCs is biologically active due to its low affinity to RBCs (Lentjes and Romijn, 1999). Thus, further *in vitro* studies are needed to elucidate the underlying mechanisms of the RBC-associated processes (e.g., adsorption and uptake) qualitatively and quantitatively.

## Data Availability

The original contributions presented in the study are included in the article/Supplementary Material; further inquiries can be directed to the corresponding author.
