# Supplementary material for: Exploring Dried Blood Spot Cortisol Concentrations as an Alternative for Monitoring Pediatric Adrenal Insufficiency Patients: A Model-Based Analysis
Source: Front Pharmacol. 2022 Mar 17;13:819590. doi: 10.3389/fphar.2022.819590 (PMC8968419; doi:10.3389/fphar.2022.819590)
Supplement: Supplementary file 1 [file DataSheet1.pdf]

Supplementary Material

1 Supplementary Data

Final model structure:

Initial conditions

$A_{depot,0} = F * DOSE$

$A_{pla,0,adult} = 0$

$A_{pla,0,child} = BASE_{child,pla} * V_c$

$A_{DBS,0,child} = 0$

$A_{p,0} = 0$

State variables and outputs

$A_{pla,adult}(t) = A_{pla}(t) + BASE_{adult} * V_c$

$A_{pla,child,pla}(t) = A_{pla}(t)$

$A_{ABASERBC} = A_{ABASEchild,DBS} - A_{ABASEchild,pla}$

$A_{DBS,child}(t) = A_{pla}(t) + A_{RBC}(t) + A_{ABASERBC}$

$K_{dA} = K_d * V_c$

$A_{max} = B_{max} * V_c$

$$A_u = \frac{A_{pla} - K_{dA} \cdot (1 + NS_{Alb}) - A_{max} + \sqrt{(A_{pla} - K_{dA} \cdot (1 + NS_{Alb}) - A_{max})^2 + 4 \cdot A_{pla} \cdot K_{dA} \cdot (1 + NS_{Alb})}}{2 \cdot (1 + NS_{Alb})}$$

$$A_p(t) = A_p(t)$$

$$C(t) = {A_{pla}(t)}/{V_c}$$

$$V_{wholeblood} = V_c + V_{delta}$$

$$C_{DBS,child}(t) = {(A_{pla}(t) + A_{RBC}(t) + A_{ABASERBC})}/{V_{wholeblood}}$$

Differential equations

$$dA_{depot}/dt = -\frac{V_{abs,max} \cdot A_{depot}}{K_{abs,50} + A_{depot}}$$

$$dA_{pla}/dt = \frac{V_{abs,max} \cdot A_{depot}}{K_{abs,50} + A_{depot}} - CL/V_c \cdot A_u - Q/V_c \cdot A_u + Q/V_p \cdot A_p$$

$$dA_p/dt = Q/V_c \cdot A_u - Q/V_p \cdot A_p$$

$$dA_{RBC}/dt = K_{aRBC} \cdot A_u - K_{aRBC} \cdot A_{RBC}$$

Estimated model parameters

$$CL_{ind} = CL_{pop} \cdot (BW/70)^{0.75} \cdot e^{\eta_{CL,ind}} \qquad \eta_{CL} \sim N(0, \omega_{CL})$$

$$V_{c,ind} = V_{c,pop} \cdot (BW/70)$$

$Q_{ind} = Q_{pop} \cdot \left({}^{BW}/_{70}\right)^{0.75}$

$V_{p,ind} = V_{p,pop} \cdot \left({}^{BW}/_{70}\right)$

$V_{abs,max} = V_{abs,max,pop} \cdot e^{\eta_{V_{abs,max}},ind}$ $\eta_{V_{abs,max}} \sim N\left(0, \omega_{V_{abs,max}}\right)$

$F_{ind} = F_{pop} \cdot e^{\eta_{F,ind}}$ $\eta_{F,ind} \sim N(0, \omega_F)$

$K_{aRBC,ind} = K_{aRBC,pop} \cdot e^{\eta_{KaRBC,ind}}$ $\eta_{KaRBC,ind} \sim N(0, \omega_{KaRBC})$

$V_{delta,children+infants,ind} = V_{delta,children+infants,pop} \cdot e^{\eta_{Vdelta,ind}}$

$V_{delta,neonates,ind} = V_{delta,neonates,pop} \cdot e^{\eta_{Vdelta,ind}}$ $\eta_{Vdelta,ind} \sim N(0, \omega_{Vdelta})$

$BASE_{adult,ind} = BASE_{adult,pop} \cdot e^{\eta_{BASE_{adult}},ind}$ $\eta_{BASE_{adult}} \sim N\left(0, \omega_{BASE,adult}\right)$

for $BASE_{child,pla}$ and $BASE_{child,DBS}$:

$BASE_{child,pop} = BASE_{observed}\ OR\ BASE_{pop,estimate}$

$BASE_{child,ind} = BASE_{child,pop} \cdot e^{\epsilon_{ind}}$ $\epsilon_{ind} \sim N(0, \sigma^2)$

Fixed model parameters

$K_{abs,50} = 4810\ nmol/L$

$F_{pop} = 1\ (-)$

$K_d = 9.71\ nmol/L$

$NS_{Alb} = 4.15\ (-)$

$B_{max} = CBG\ concentration\ \left[{}^{nmol}/_{L}\right] = 22.4 \frac{ug}{mL} * \frac{1000}{52 \frac{g}{nmol}} \left[{}^{nmol}/_{L}\right]\ when\ not\ measured$

NONMEM model code

```
$PROBLEM
pediatric plasma and DBS cortisol PK model

$INPUT
ID COHORT DROP=DAT2 DROP=TIME2 TIME AMT DROP=RATE DROP=DVX
DROP=LDVX DROP=DV2 DROP=LDV2 DROP=DV3 DV ODV DROP=MDVX DROP=MDV2
MDV EVID BLQ CMT FLAG FLAGM FLAGB HCT HCTF AGE BW HT BMI BSA SEX CBG
ALB DOSE TAFO

;ignore adult iv data
$DATA  dataset.csv IGNORE=@ IGNORE(BLQ.EQ.1) IGNORE(FLAG.EQ.10)

$SUBROUTINE ADVAN13 TOL=9

$MODEL
COMP = (DEPOT DEFDOSE)                 ;dose compartment
COMP = (CENTRAL DEFOBSERVATION)        ;central plasma compartment
COMP = (PERIPH)                        ;peripheral plasma compartment
COMP = (OUTPUT1)                       ;compartment with cortisol associated to RBCs

$PK

;allometric scaling applied to CL and Q (exponent 0.75) and to V1 andV2 (exponent 1),
;referring to 70 kg as normal adult BW

TVCL = THETA(1)*((BW/70)**0.75)
CL = TVCL*EXP(ETA(1))

TVV1 = THETA(2)*(BW/70)
V1 = TVV1*EXP(ETA(2))

TVQ = THETA(3)*(BW/70)**0.75
Q = TVQ*EXP(ETA(3))

TVV2 = THETA(4)*(BW/70)
V2 = TVV2*EXP(ETA(4))

TVKM = THETA(5)
KM = TVKM*EXP(ETA(5))

;number of CBG binding sites fixed to 1
TVBS = THETA(6)
```

```
BS = TVBS*EXP(ETA(6))

TVKD = THETA(7)
KD = TVKD*EXP(ETA(7))

;converting concentration to amount
KDa = KD*V1

TVNSALB = THETA(8)
NSALB = TVNSALB*EXP(ETA(8))

TVVM = THETA(9)
VM = TVVM*EXP(ETA(9))

;CBG given in dataset for adult study 2 and for pediatric data
CBG2 = CBG
IF(CBG.EQ.-99) THEN
CBG2 = THETA(15)
ENDIF

;converting CBG from ug/mL to nmol/L. MW=52000g/mol
CBGmol = CBG2*1000/52

BMAX = CBGmol*BS
;converting concentration to amount
AMAX = BMAX*V1

TVF1 = THETA(10)
F1 = TVF1*EXP(ETA(10))

TVKaRBC = THETA(11)
KaRBC = TVKaRBC*EXP(ETA(11))

;adult (COHORT 4) baseline
IF (COHORT.EQ.4) THEN
TVABASE = THETA(12)
ABASE = TVABASE*EXP(ETA(12))
ELSE
ABASE = 0
ENDIF

;Vdelta for neonates (COHORT 3) and children+infants (COHORT 1+2)
IF(COHORT.LT.3) THEN
TVV3 = THETA(13)
ENDIF
IF(COHORT.EQ.3) THEN
TVV3 = THETA(16)
ENDIF
```

```
V3 = TVV3*EXP(ETA(14))

IF(COHORT.EQ.4) THEN
IBASE =0
IBASEB = 0
ENDIF

;pediatric baseline observations in plasma (FLAGM=0)
IF(COHORT.LT.4.AND.FLAGM.EQ.0) THEN
OBASE = ODV
ENDIF
;pediatric baseline observations in DBS (FLAGM=1)
IF(COHORT.LT.4.AND.FLAGM.EQ.1) THEN
OBASEB = ODV
ENDIF

;pediatric baseline in plasma if no baseline observation given
IF(OBASE.EQ.0) THEN
IBASE = THETA(17)*EXP(ETA(13)*THETA(14))
ENDIF

;pediatric baseline in plasma if baseline observation given
IF(OBASE.GT.0) THEN
IBASE = OBASE*EXP(ETA(13)*THETA(14))
ENDIF

IF(COHORT.EQ.4) THEN
IBASE = 0
ENDIF

;pediatric baseline in DBS if no baseline observation given
IF(OBASEB.EQ.0) THEN
IBASEB = THETA(18)*EXP(ETA(13)*THETA(14))
ENDIF
;pediatric baseline in DBS if baseline observation given
IF(OBASEB.GT.0) THEN
IBASEB = OBASEB*EXP(ETA(13)*THETA(14))
ENDIF

IF(COHORT.EQ.4) THEN
IBASEB = 0
ENDIF

; Time after dose
TAD = 0
IF (AMT.GT.0) THEN
TDOS = TIME
```

```
TAD = 0.0
ENDIF
IF (AMT.EQ.0) TAD = TIME-TDOS
IF (TAD.LT.0) TAD = 0

S1 = V1
k10 = CL/V1
k12 = Q/V1
k21 = Q/V2

;amount at timepoint 0 in central plasma compartment, IBASE is 0 for adults
A_0(2) = IBASE*V1

;baseline amounts
AIBASE = IBASE*V1
AIBASEB = IBASEB*(V1+V3)

ABASERBC = AIBASEB-AIBASE
IF(ABASERBC.LT.0) THEN
ABASERBC = 0.1
ENDIF

$DES
;calculating unbound amount in plasma with binding model from Melin et al., 2017
AUP = (A(2)-KDa*(1+NSALB)-AMAX+SQRT(((A(2)-KDa*(1+NSALB)-
AMAX)**2)+4*KDa*A(2)*(1+NSALB)))/(2*(1+NSALB))

DADT(1) = -(VM*A(1)/(KM+A(1)))              ;saturable absorption from dose compartment
DADT(2) =  (VM*A(1)/(KM+A(1))) - k10*AUP - k12*AUP + k21*A(3) ;central compartment
DADT(3) = -k21*A(3)+k12*AUP                 ;peripheral compartment
DADT(4) = AUP*KaRBC-A(4)*KaRBC              ;compartment with cortisol associated to RBCs


$ERROR

A1 = A(1)
A2 = A(2)
AUP2 = (A(2)-KDa*(1+NSALB)-AMAX+SQRT(((A(2)-KDa*(1+NSALB)-
AMAX)**2)+4*KDa*A(2)*(1+NSALB)))/(2*(1+NSALB))
A3 = A(3)
A4 = A(4)
C1 = A1/V1
C2 = A2/V1

BASEB = 0
BASEP = 0
IPRED = 0
```

```
;TAFO = time at baseline observation
IF(TAFO.EQ.0) THEN
BASEB = IBASEB
BASEP = IBASE
ENDIF

;pediatric DBS concentrations
IF (FLAGM.EQ.1.AND.COHORT.LT.4) THEN
IPRED = (A2+A4+ABASERBC)/(V1+V3)
ENDIF

;pediatric plasma concentrations
IF (FLAGM.EQ.0.AND.COHORT.LT.4) THEN
IPRED = A2/V1
ENDIF

;adult plasma concentrations
IF(COHORT.EQ.4) THEN
IPRED = A2/V1 + ABASE
ENDIF

IF(IPRED.GT.0) THEN
IPRED = LOG(IPRED)
ELSE
IPRED = LOG(IPRED+0.01)
ENDIF
W = THETA(14)
Y = IPRED+W*EPS(1)
IRES = DV-IPRED
IWRES = IRES/W


$THETA
(0.001,478.556342893741)        ; 1. CL [L/h]
(0.001,10.7708078998368)        ; 2. V1 [L]
(0.001,297.932200383505)        ; 3. Q [L/h]
(0.001,154.39641074985)         ; 4. V2 [L]
4810 FIX                        ; 5. Km [nmol] fixed to final model estimate from Michelet et
al., 2020
1 FIX                           ; 6. BS [-], from binding model in Melin et al., 2017
9.71 FIX                        ; 7. KD [nmol/L], from binding model in Melin et al., 2017
4.15 FIX                        ; 8. NSALB [-], from binding model in Melin et al., 2017
(0.001,18048.2801258582)        ; 9. Vmax [nmol/h]
1 FIX                           ; 10. Bioavailability [-]
(0.001,3.36024393996957)        ; 11. KaRBC [-], association constant for red blood cells
(0.001,14.1273301672484,100)    ; 12. adult baseline [nmol/L]
(0.001,0.518880215219603)       ; 13. Vdelta children+infants [L]
```

```
(0.001,0.143723649120103)          ; 14. additive error on a logarithmic scale [sd]
22.4 FIX                           ; 15. CBG Baseline [ug/mL]
(0.001,0.547320889482403)          ; 16. Vdelta neonates [L]
(0.001,14.2481176260859)           ; 17. pediatric plasma baseline [nmol/L]
(0.001,20.2496941719316)           ; 18. pediatric DBS baseline [nmol/L]

$OMEGA
0.0564712567464341     ;1. CL
0  FIX                 ;2. V1
0  FIX                 ;3. Q
0  FIX                 ;4. V2
0.208                  ;5. KM
0.049  FIX             ;6. BS
0  FIX                 ;7. KD
0  FIX                 ;8. NSALB
0.351586646476232      ;9. Vmax
0.149665511339003      ;10. Bioavailability
0  FIX                 ;11. KaRBC
0.115109316120945      ;12. adult baseline
1  FIX                 ;13. pediatric baselines
0.183683503993367      ;14. Vdelta

$SIGMA  1  FIX

$ESTIMATION MAXEVAL=10000 METHOD=1 INTER SIG=2 NOABORT
$COVARIANCE UNCONDITIONAl PRINT=E

$TABLE ID TIME TAD TAFO DV PRED IPRED WRES IWRES CWRES COHORT MDV A1 A2
A3 A4 AUP C1 C2 FLAGM FLAGB ONEHEADER NOPRINT FILE=sdtab230

$TABLE ID TIME DV CL V1 Q V2 V3 KaRBC IBASE IBASEB BMAX KD NSALB VM KM
OBASE OBASE ABASE COHORT F1 CBG BW HT ALB AGE ODV FLAGM FLAGB ETA1
ETA2 ETA3 ETA4 ETA5 ETA6 ETA7 ETA8 ETA9 ETA10 ETA11 ETA12 ETA13 ETA14
NOAPPEND ONEHEADER NOPRINT FILE=patab230

$TABLE DV PRED IPRED WRES IWRES CWRES MDV NPDE ESAMPLE=1000
SEED=1234567 ONEHEADER NOPRINT FILE=NPDEtab_230
```

2 Supplementary Figures

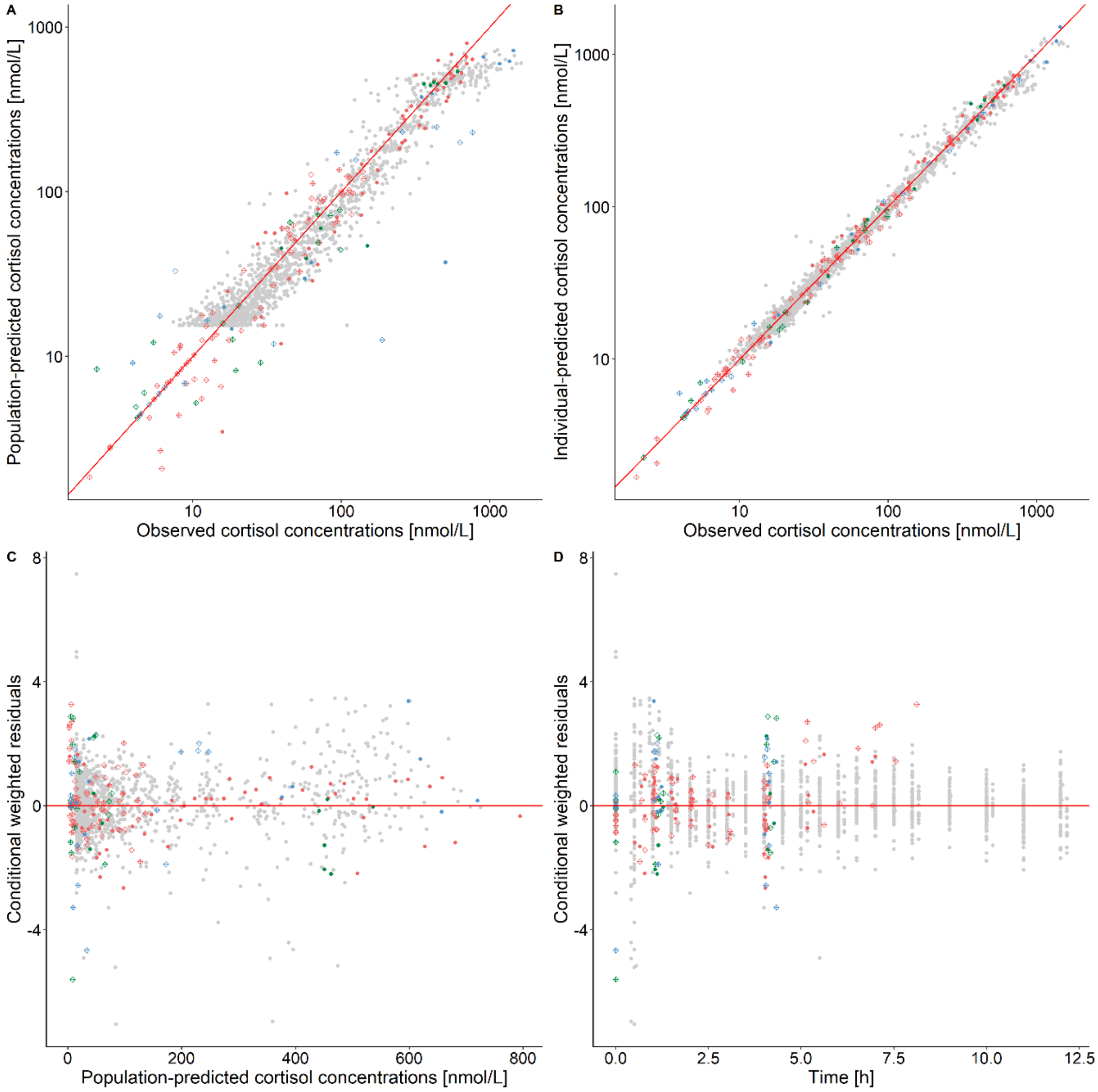

Supplementary Figure 1. Goodness-of-fit plots of the developed cortisol pharmacokinetic (PK) model.
(A) Population-predicted cortisol concentrations versus observed cortisol concentrations, **(B)** Individual cortisol predictions versus observed cortisol concentrations, **(C)** Conditional weighted residuals versus population-predicted cortisol concentrations, **(D)** Conditional weighted residuals versus time. Red: children, green: infants, blue: neonates, gray: adult observations, filled circles: plasma concentrations, diamonds: dried blood spot concentrations, red line: line of identity (A, B), line y=0 (C, D).

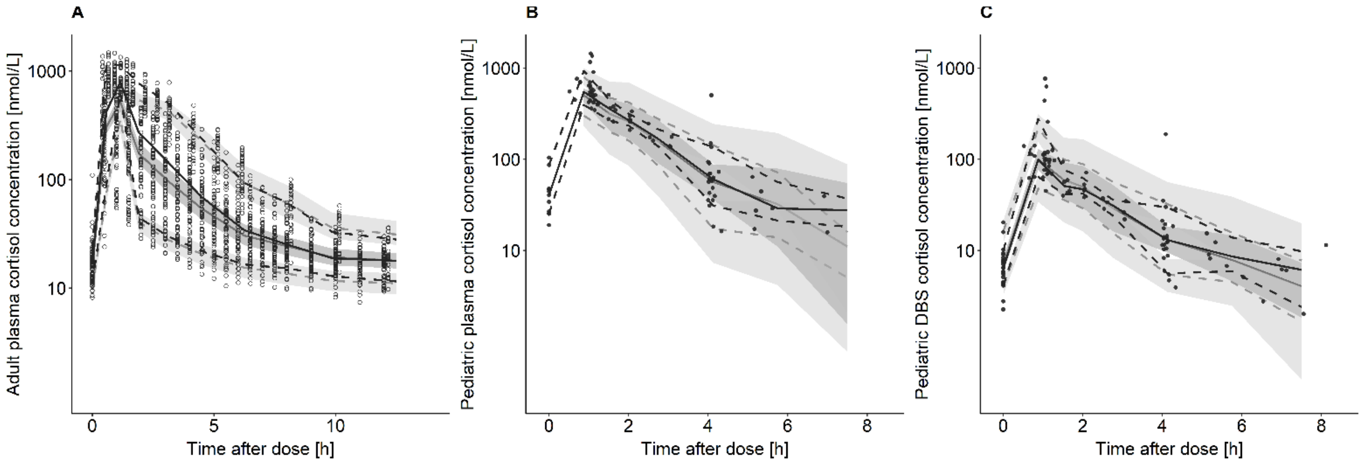

Supplementary Figure 2. Visual predictive check (n=1000 simulations) for developed cortisol PK model. Adult total cortisol plasma concentrations **(A)**, pediatric total cortisol plasma concentrations **(B)**, pediatric total cortisol dried blood spot (DBS) concentrations **(C)**.
Circles: cortisol observations, black/gray solid line: 50th percentile of observed/simulated concentrations, black/gray dashed lines: 10th and 90th percentiles of observed/simulated concentrations, gray shaded areas: 95 % confidence intervals for the percentiles of the simulated data.

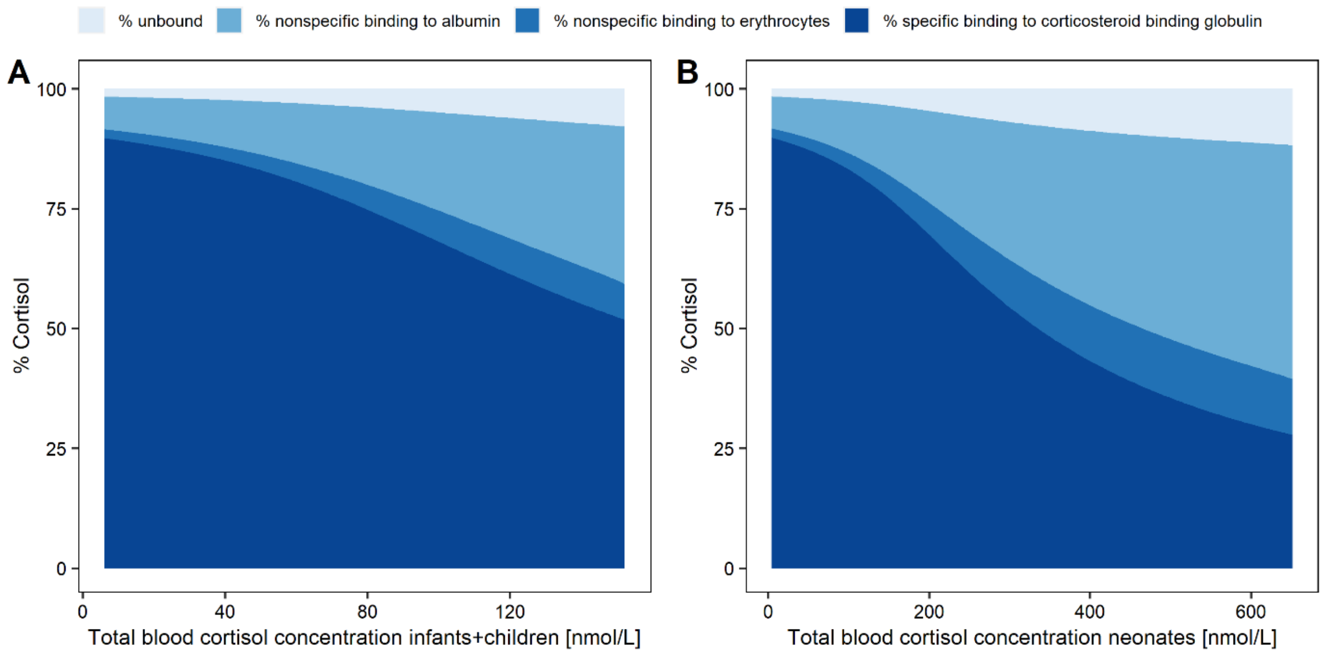

Supplementary Figure 3. Simulated cortisol concentration fractions (%) after dosing with 4 mg hydrocortisone over total whole blood (dried blood spot) concentration (LLOQ=1.8 nmol/L to Cmax) in infants and children **(A)** and neonates **(B)**. Unbound (pale blue), with nonspecific linear binding to albumin (light blue), nonspecific linear binding to red blood cells (middle blue) and specific nonlinear binding (dark blue) to corticosteroid binding globulin.
